# Supplementary material for: Clusters of science and health related Twitter users become more isolated during the COVID-19 pandemic
Source: Sci Rep. 2021 Oct 4;11:19655. doi: 10.1038/s41598-021-99301-0 (PMC8490394; doi:10.1038/s41598-021-99301-0)
Supplement: Supplementary file 1 — Supplementary Information. [file 41598_2021_99301_MOESM1_ESM.pdf]

Clusters of science and health related Twitter users become more isolated during the COVID-19 pandemic

Francesco Durazzi *†
Department of Astronomy and Physics (DIFA)
University of Bologna
Bologna 40127, Italy
francesco.durazzi2@unibo.it

Martin Müller *
Digital Epidemiology Lab
Ecole polytechnique fédérale de Lausanne (EPFL)
1202 Geneva, Switzerland
martin.muller@epfl.ch

Marcel Salathé
Digital Epidemiology Lab
Ecole polytechnique fédérale de Lausanne (EPFL)
1202 Geneva, Switzerland
marcel.salathe@epfl.ch

Daniel Remondini
Department of Astronomy and Physics (DIFA)
University of Bologna
Bologna 40127, Italy
daniel.remondini@unibo.it

September 13, 2021

*F.D. and M.M. contributed equally to this work.
†To whom correspondence may be addressed. Email: francesco.durazzi2@unibo.it

1 Supplementary Text

1.1 Data collection

Collection started on January 13, 2020 a few weeks after first reports about a disease outbreak in Wuhan, China surfaced. Throughout the collection period, the keywords were changed in order to accommodate for the various ways the virus was referred to (see table 2). Initially the virus was referred to as "wuhan virus" and later as 2019-nCoV (2019 novel coronavirus). On February 11 the ICTV (International Committee on Taxonomy of Viruses) changed the official name to sars-cov-2 and COVID-19, for the virus and the disease respectively.

Due to the high volume of data small interruptions occurred during data collection when no data was collected. Four interruptions were for longer than one hour, the longest being 9 hours on April 11.

1.2 Geo-localization of tweets

In order to geo-localize a tweet the following procedure was performed:

1. Geo coordinates ($\sim 0.1\%$ of original tweets in dataset): Tweet contains coordinates (longitude and latitude) information.

2. Place (2.9%): Users can tag a tweet with a named place. Tweets with place indication contain structured geo information, including a geographical bounding box.

3. Parsable user location (61.9%): We use the Python library `local-geocode`[1] in order to parse the user location field. This field contains unstructured text and may reference one or multiple places and/or countries. It also sometimes contains humorous or imaginary places (e.g. "the end of the universe"). The `local-geocode` library makes use of the geonames database and performs substring matching against place names in this database in order to obtain structured geographical information (also known as geocoding). In the matching, only places with a population larger than 30k are considered. `local-geocode` has been compared against `geopy`[2] (using the Nominatim library), which is frequently used for this task. Visual inspections of the country-level disagreements between both tools, indicate that `local-geocode` generally performs better in this task. This is likely due to the fact that `local-geocode` only considers relatively well known places, therefore ignoring imaginary names whereas geopy attempts to provide a (wrong) result in these cases. However, human-level benchmarking would need to be conducted in order to come to a final conclusion on the performance of both tools for Twitter user location decoding.

1.3 Network analysis

Community detection. We applied Louvain's community detection algorithm (implemented in Python's Networkit package, PLM function), setting the default resolution parameter $\gamma = 1$. Since each run of the algorithm produces different results, we run the algorithm for 50 trials and assigned each user to the community it was mostly found into. On average, about 15 communities reached a size larger than 10^5 (15.42 ± 0.09) (see Supplementary Fig. 2). In order to assign each user to a community, we counted how many times each node appeared in the same community along the 50 trials (the same community was hypothesized to be that of maximal overlap within all trials). The ratio of times each node was found in the same community was used as a 0-1 score ("community score") about goodness of identification of the community associated to each node (Supplementary Fig. 6). The average community score computed on all the users is 0.92(S.D. = 0.10).

Stability over time. Furthermore, we analyzed the overlap of user IDs in communities obtained from the full network and from networks reconstructed with data aggregated per month. We call temporary communities the ones detected in the monthly time-windows and cumulative communities the ones detected on the full time-aggregated network. Retweets posted during January and February were lower than the rest of observational period, so we joined the two months into a single time-window. This means that four temporary networks were built aggregating the retweets sent during January-February, March, April, May 2020 separately. A fair stability over time was observed overall (see Supplementary Fig. 8). Temporal stability was highest for the largest communities (labelled from A to H), having an average

[1] `https://github.com/mar-muel/local-geocode`
[2] `https://github.com/geopy/geopy`

overlap of 72% (min 44%, max 94%) with the most overlapping temporary communities. Also smaller communities, in particular L, M, and J, showed a fair temporal stability (avg. overlap 57%, min 20%, max 89%).

In Supplementary Fig 9, we plot the fluxes of users between temporary communities. In general, the fraction of user being conserved between consecutive time-windows tend to be assigned to the same network community, with only minor inter-communities dynamics. Some cumulative communities, such as A and E from the Other super-community, were divided into multiple sub-groups in some time-windows, confirming the difficulty of finding a clear identity for these communities. The National elites communities emerge only in some time-windows, possibly in correspondence with key events in the respective countries. Most of the users from the major Political communities (C, F, H) do not move to another community over time. All the time-windows show a small flux of users from the International sci-health community B toward the other communities. This out-flux from B is majorly fragmented in the time period from February to March, while since April the major out-flux from B is toward the Political community C.

Top users characterization. The network's communities are composed by users with different roles and centrality inside the network. For a finer characterization of the authorities of this retweet network, we selected as top users the 1000 most retweeted users for each super-community. We computed well-known centrality measures, with the Python's package Networkit, and show their correlation in Supplementary Figure 11. The node betweenness centrality for each user in the network was estimated considering the shortest paths between 100k randomly sampled nodes. Correlation between centrality measures do not display different patterns for different super-communities. Out-degree and in-degree have the meaning of the number of retweets respectively received and sent. The distribution of received retweets is centered on the highest value for the Political super-community, meaning more attention received. Clustering coefficient is centered on the lowest value for Other, meaning a sparser and less modular community.

Sentiment analysis. To investigate the association between tweets content and the changes of the retweet volume for each super-community we performed a sentiment analysis through the Python's library TextBlob, which operates a lexicon based analysis of the text to compute the subjectivity and the polarity of a message. In Supplementary Figure 12 and 13 we show respectively the average subjectivity score and polarity score of the original tweets written by each super-community. We then classified each retweet in the dataset as Negative, Neutral or Positive depending on whether the shared tweet had polarity less than -1/3, between -1/3 and +1/3 or higher than 1/3 respectively. We showed the proportion of each sentiment category over-time in Supplementary Figure 14. The analysis did not reveal any significant time trend, neither average differences between the super-community, neither in terms of subjectivity or polarity.

The same result was observed by considering only the subset of original tweets written by the top 1000 users for each super-community. On the same subset of tweets, we also performed a Deep Learning classification with a roBERTa classifier, but again we did not observe any change over time.

2 Supplementary Figures

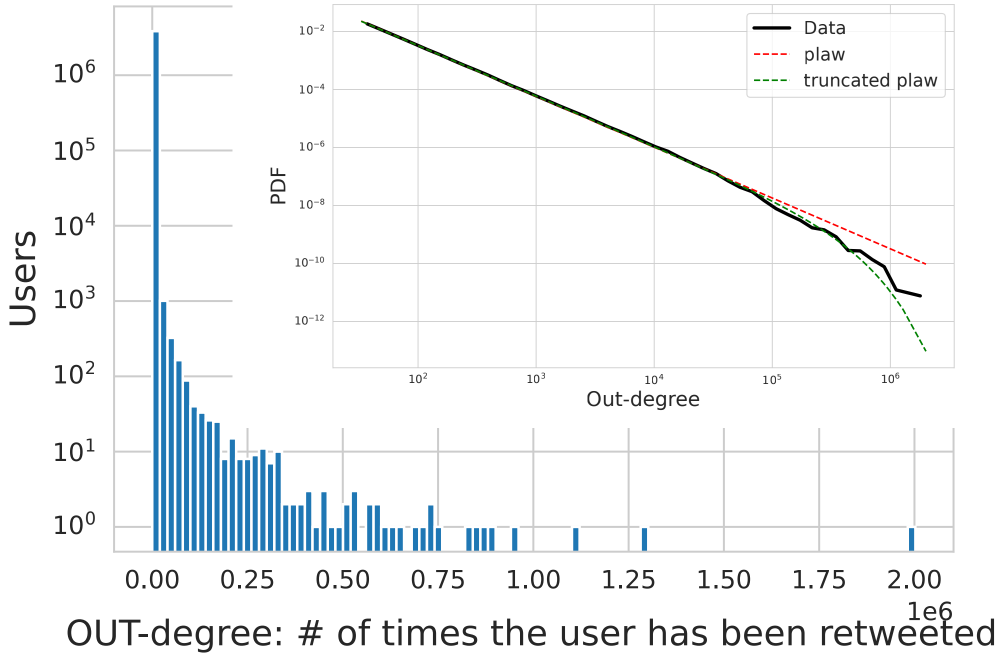

Figure 1: **Weighted out-degree of the retweet network.** Inbox: log-log plot of the probability density function of the weighted out-degree. The distribution can be fitted through a exponentially truncated power-law, written as $x^{-\alpha}e^{-\lambda x}$ with $\alpha = -1.75$, $\lambda = 3.5 \times 10^{-6}$. The loss of linearity for high degree nodes is probably due to an under-sampling of the retweets received from viral tweets, due to the limitations imposed by Twitter on data collection (see Methods).

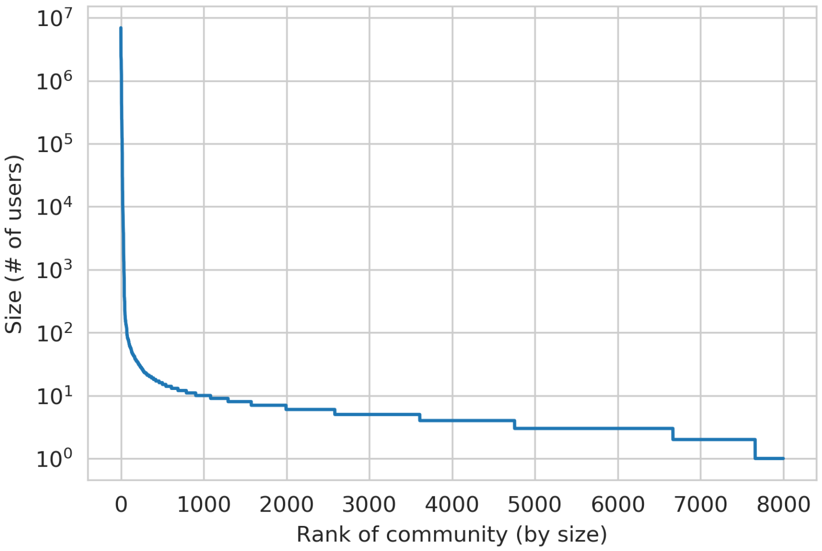

Figure 2: **Typical distribution of community size.** Size of the communities obtained within one single run of Louvain's community detection algorithm.

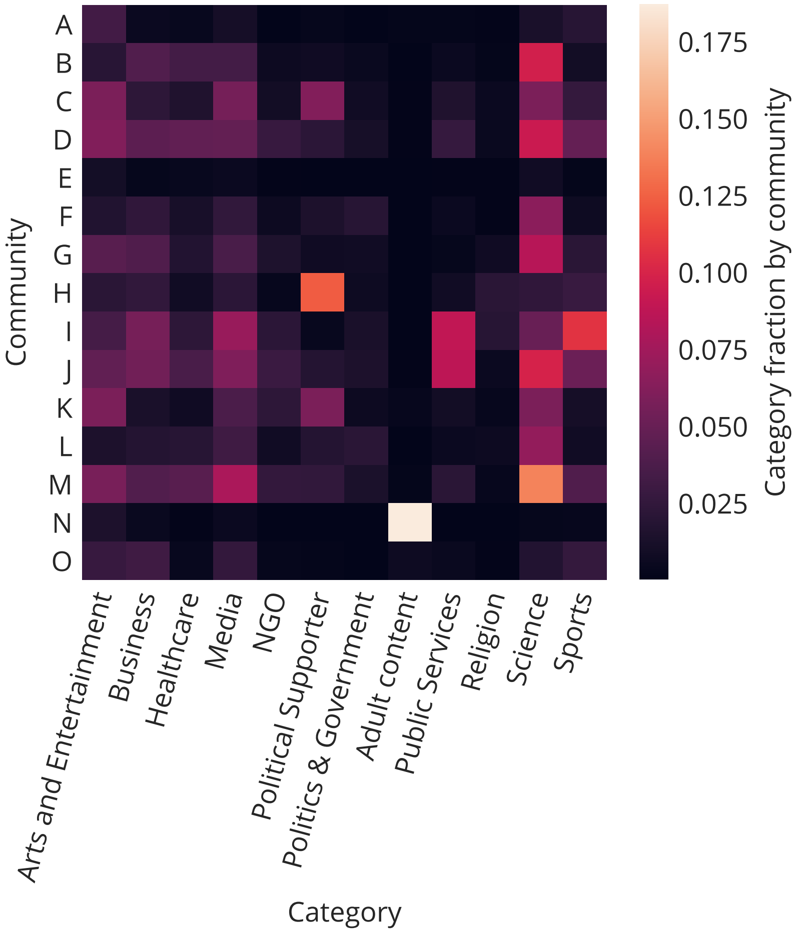

Figure 3: **Heatmap of category fraction by community.** Category "Other" is the largest fraction in all communities but not shown in the figure.

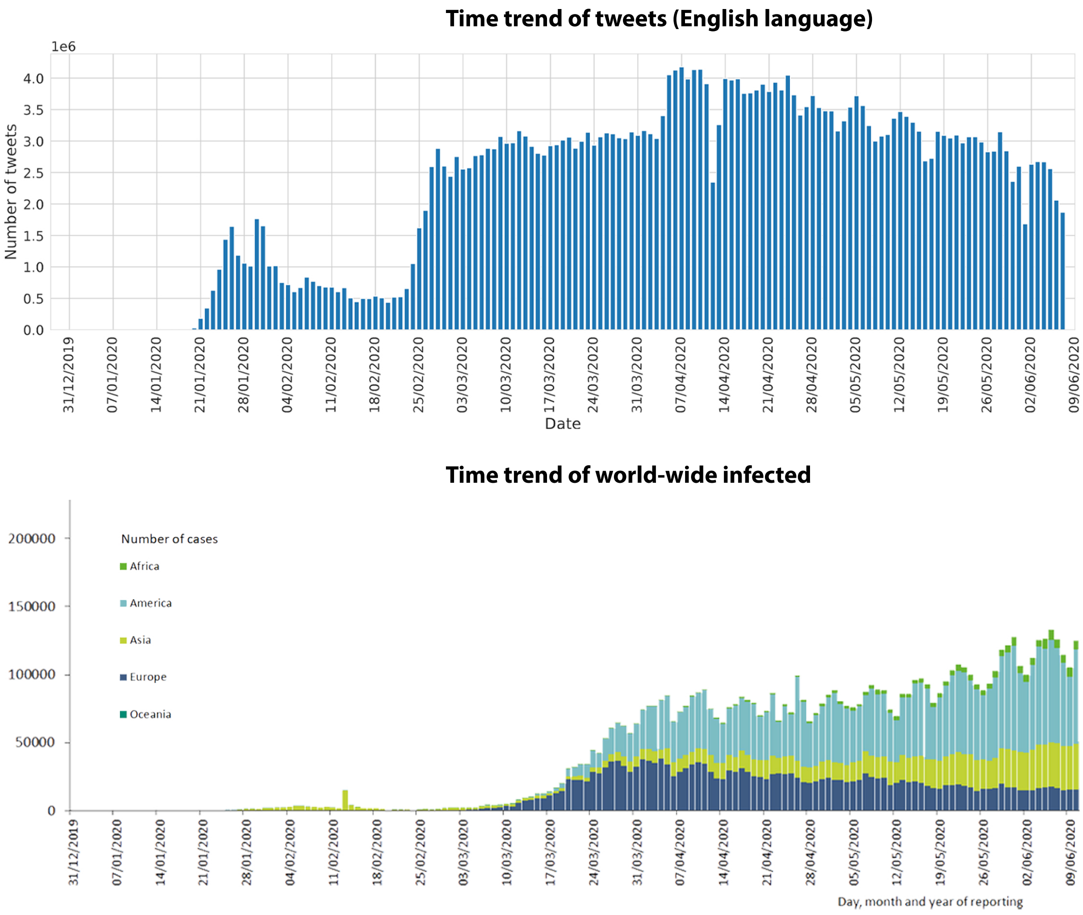

Figure 4: **Number of daily tweets and daily COVID-19 cases**. Top: count of tweets collected daily during the period we observed. Both original tweets and retweets are counted. Bottom: distribution of COVID-19 cases worldwide (website: `https://www.ecdc.europa.eu/en/geographical-distribution-2019-ncov-cases`).

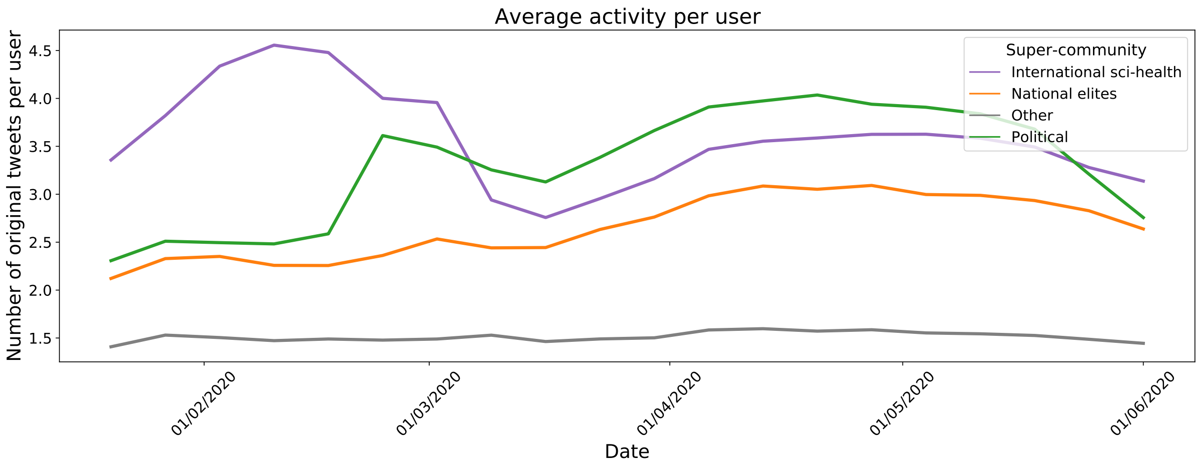

Figure 5: **Average activity per user.** Number of weekly original tweets written by the users of each super-community, divided for the number of active users in that week from each super-community.

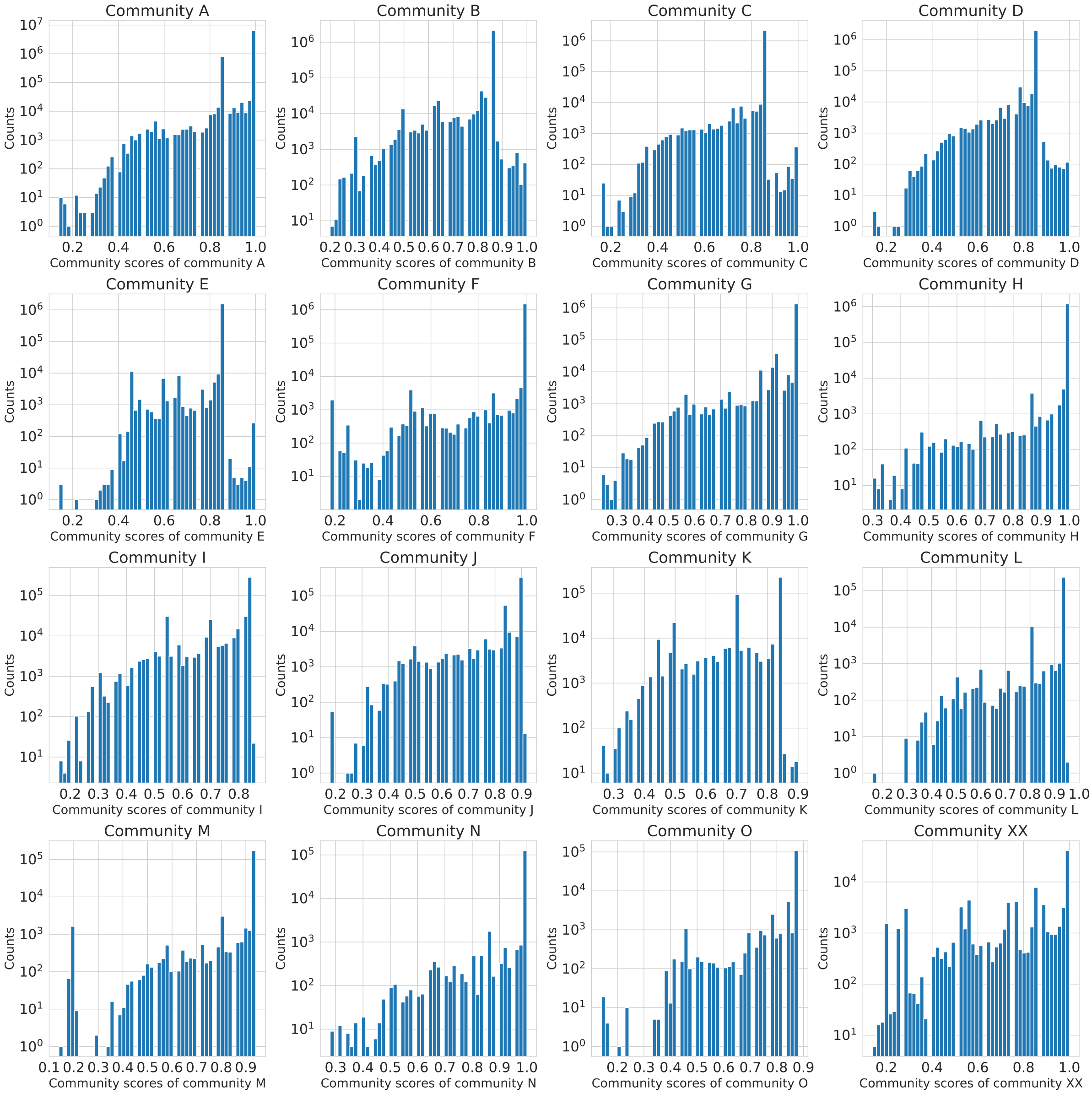

Figure 6: **Community score for the 15 largest communities.** Score distribution is shown in semilogarithmic scale: the majority of nodes have a score very close to 1. Community XX includes all the users that do not belong to communities A-O for most of the algorithm stochastic repetitions.

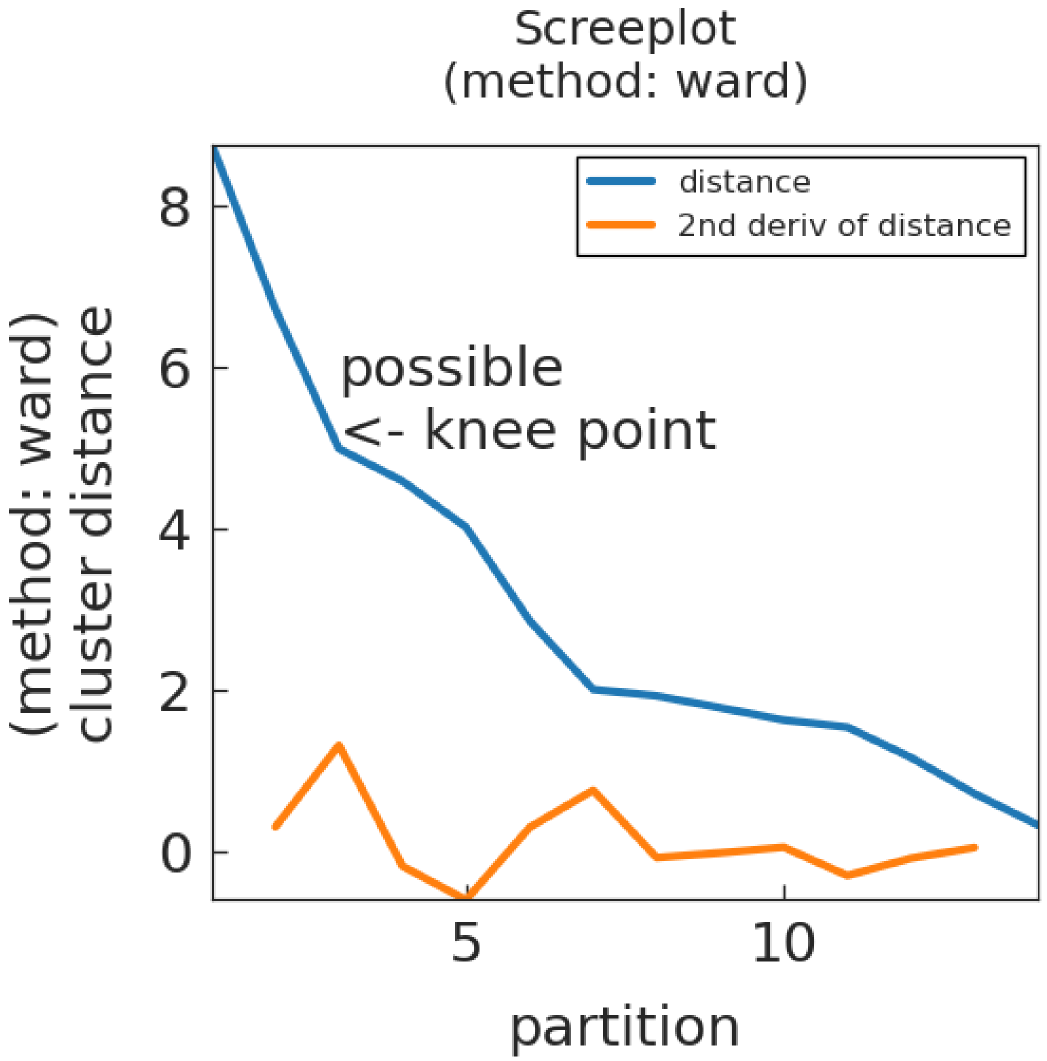

Figure 7: **Average inter-clusters distance computed on user category arrays.** The blue line is the average distance between the clusters of communities as a function of the number of clusters. Each community is represented by a vector of category abundances as in Figure 2. The orange line is the 2nd order derivative of the distance, to individuate the knee point. The calculated knee point for the distance is 3 clusters.

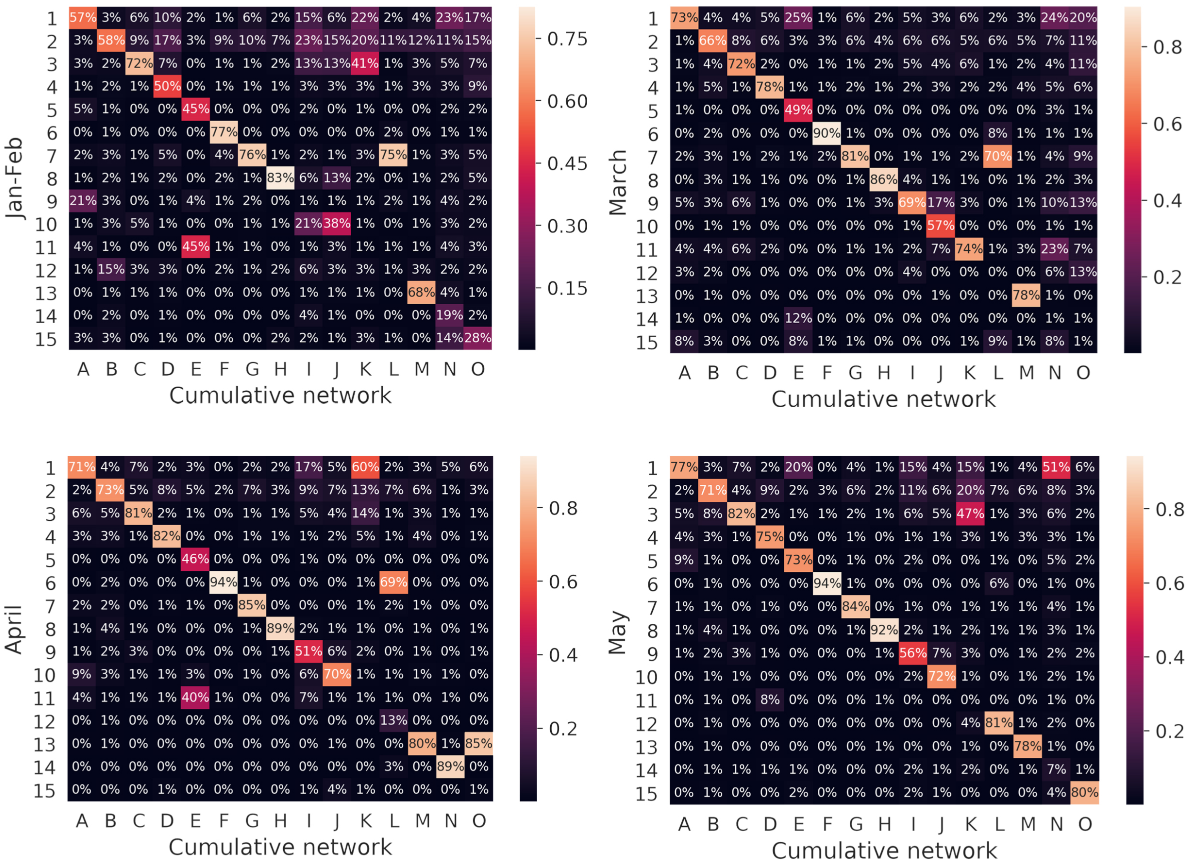

Figure 8: **Stability of the communities over time.** Overlap of communities detected on the aggregated network (cumulative communities) with respect to communities detected on four time-windows (temporary communities). Each column shows how each cumulative community (x-axis) was distributed through the temporary communities (y-axis). For each heatmap, percentages are computed respect to the total number of users in that time window.

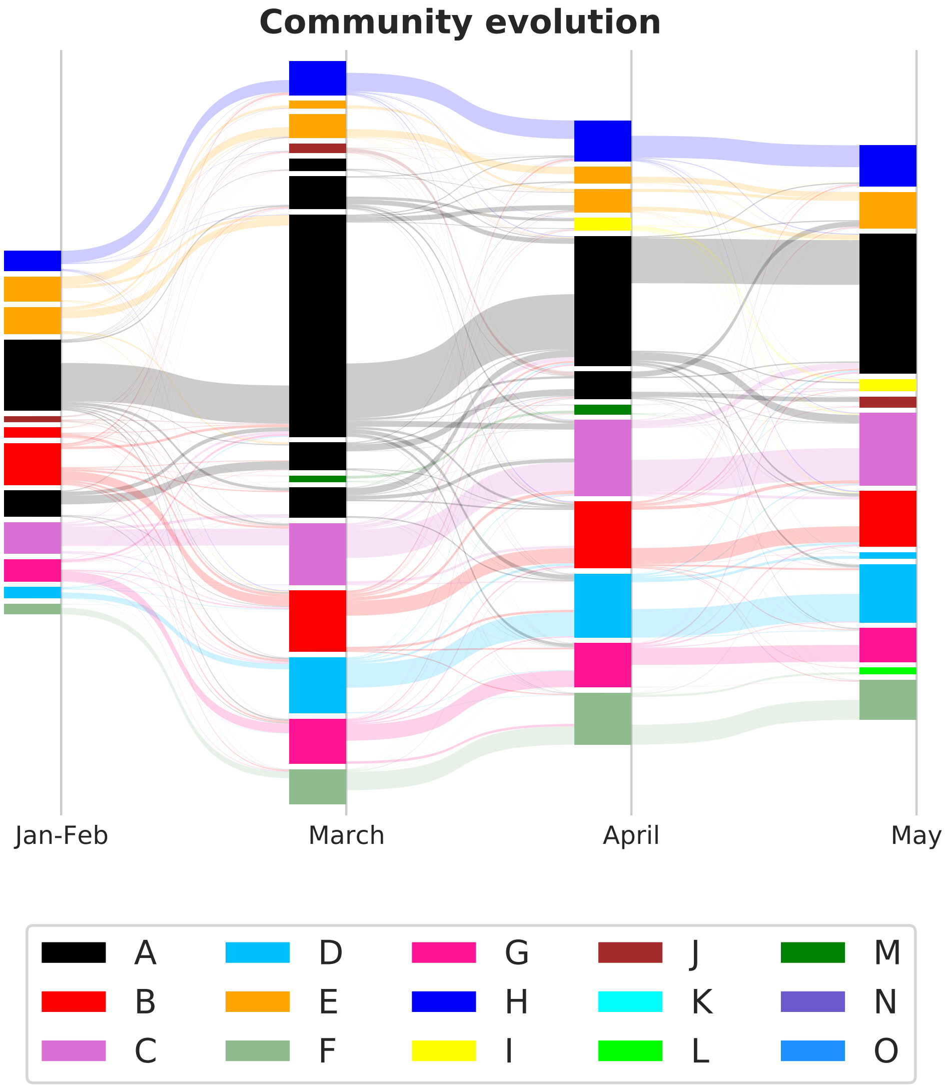

Figure 9: **Alluvial plot of the time-evolution of the temporary network communities.** The communities were detected separately on four time-windows, following the same approach of the time-aggregated network. For the plot, a group of 200K users (1%) was randomly sampled. Each temporary community (rectangle) was coloured as the time-aggregated community with which it overlapped the most. The size of each rectangle is proportional to the number of users assigned to each community. The size of the alluvial fluxes is proportional to the number of users moving from one temporary community to another in the next time-window.

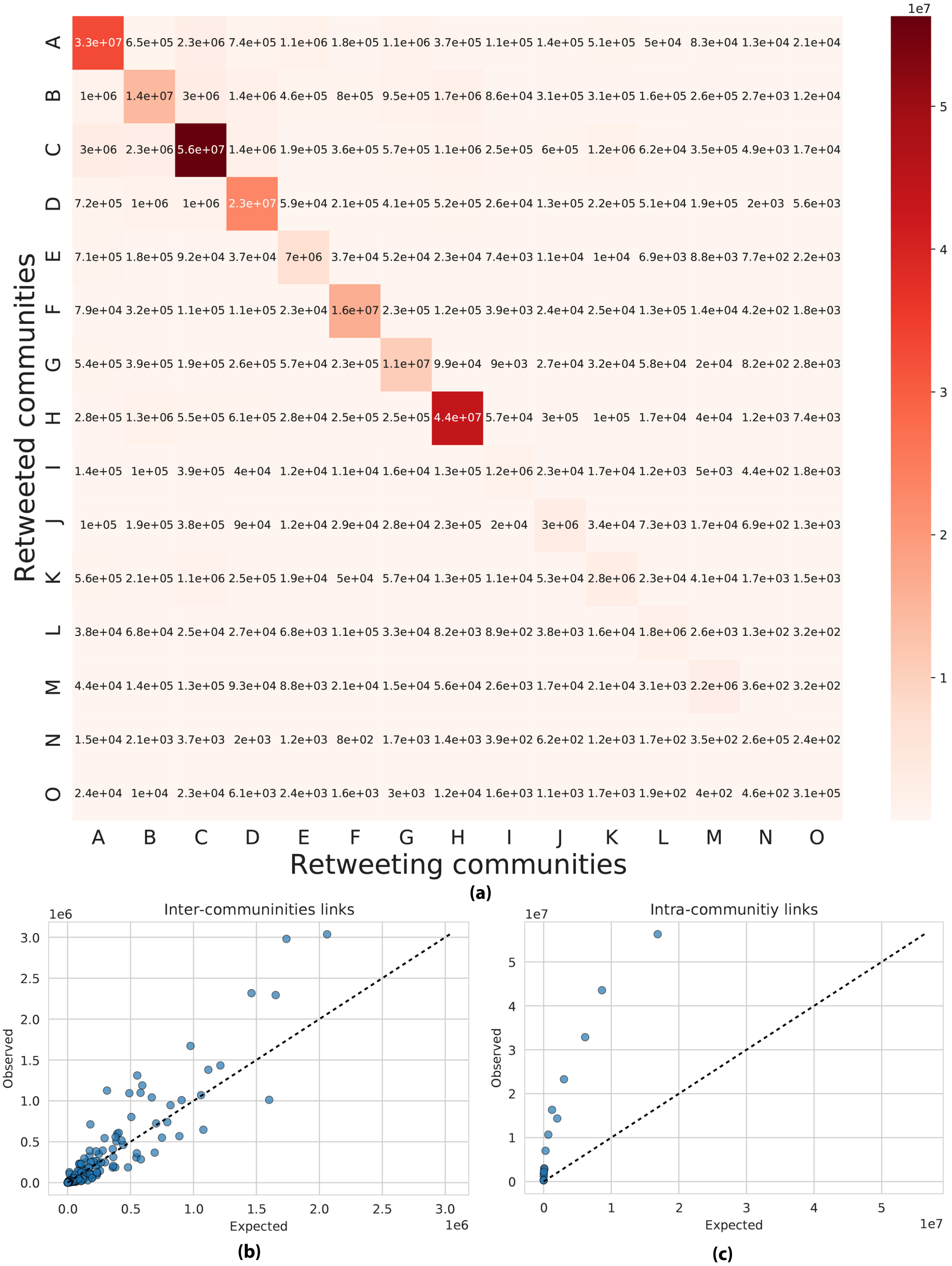

Figure 10: **Mixing matrix of the retweet network.** (a) Mixing matrix of the network, obtained by collapsing all the users belonging to a community into a single node. (b-c) Scatter plot of observed links and expected number of links assuming a random mixing null model between communities. (b) Inter-community links, without considering intra-community retweets. (c) Intra-community links. Communities of users, by construction, have more intra-links than expected by a random mixing null model.

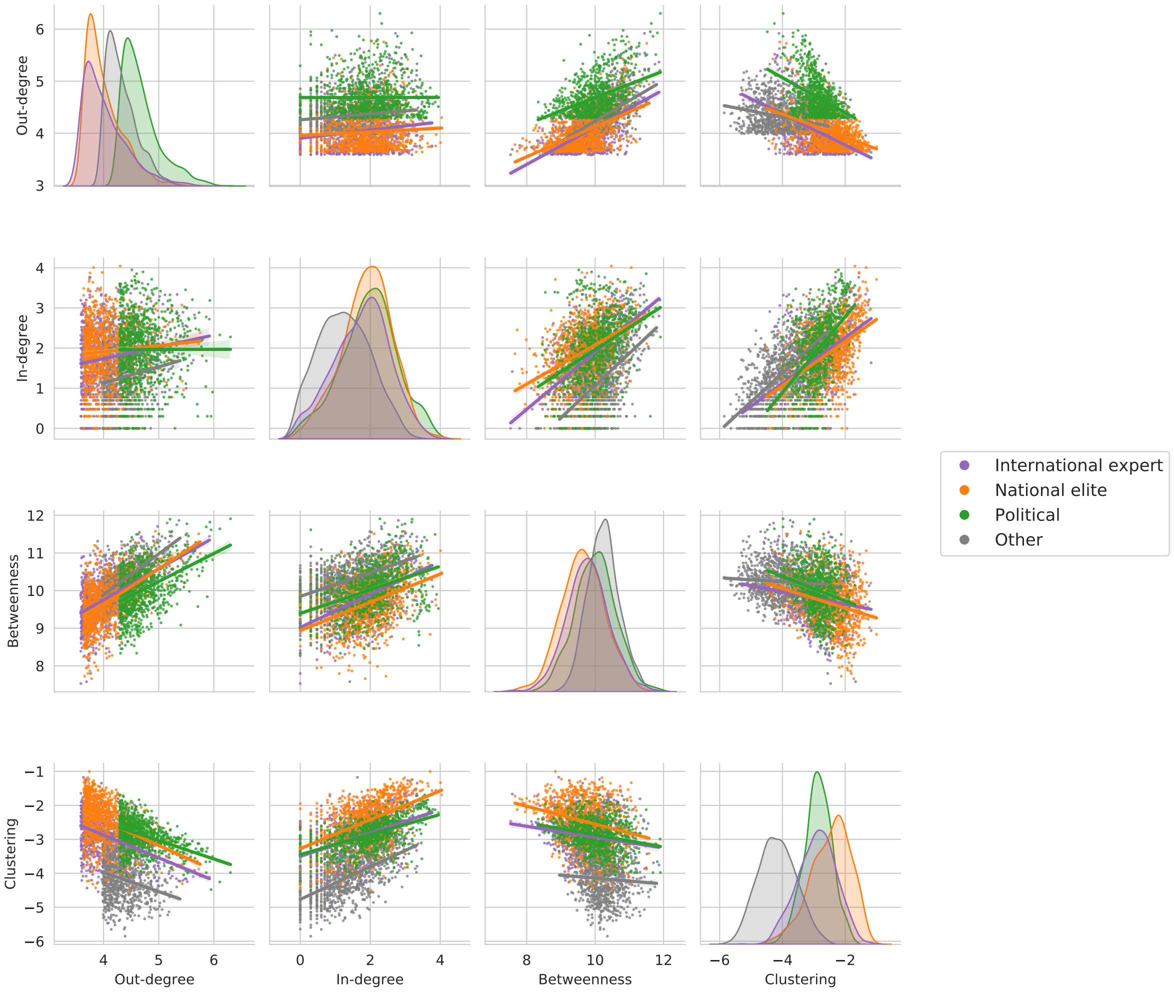

Figure 11: **Network centrality measures.** Centrality measures of the top users in each super-community, portrayed in log-log scale. Top users are chosen as the most retweeted 1000 for each super-community.

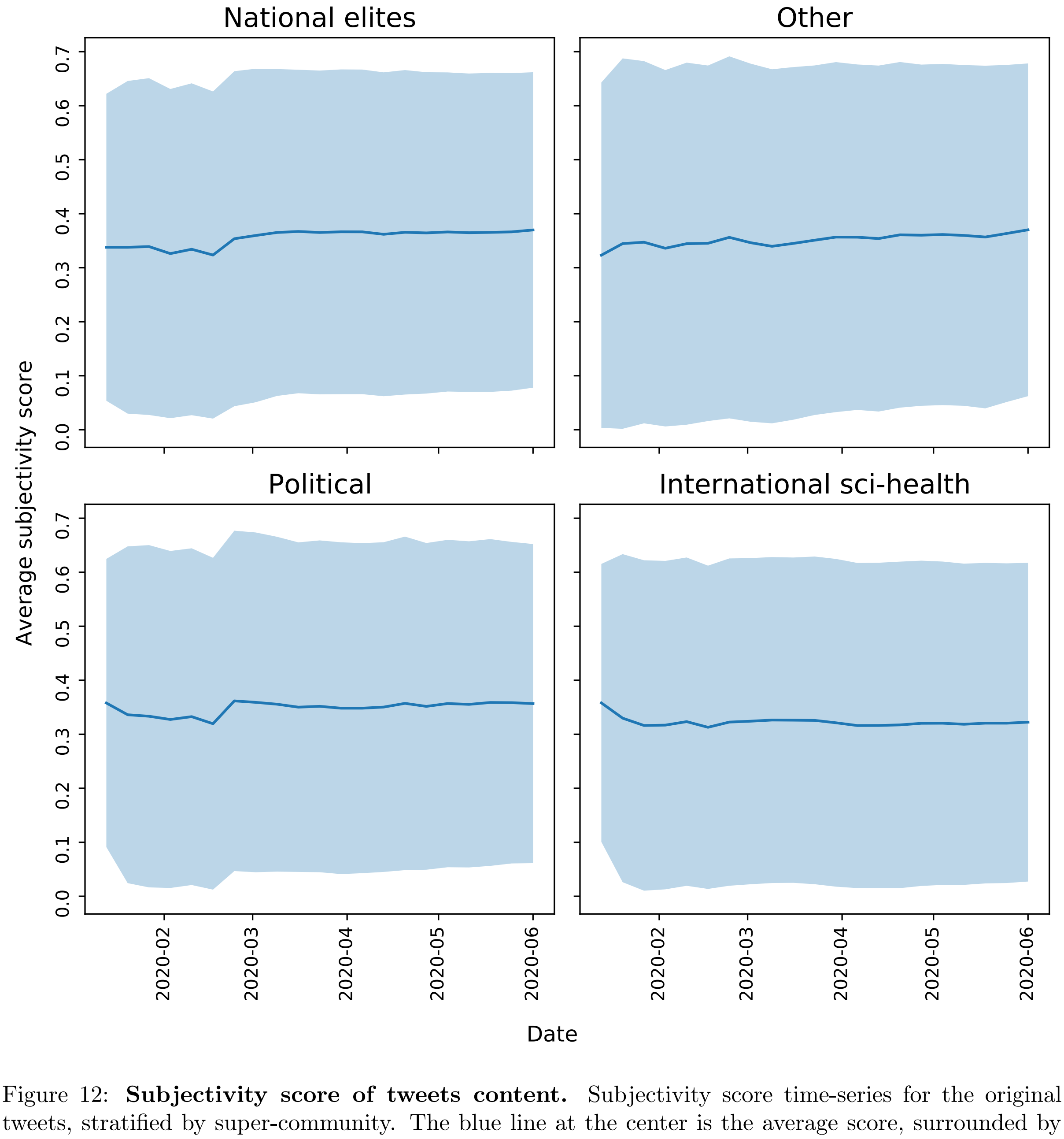

Figure 12: **Subjectivity score of tweets content.** Subjectivity score time-series for the original tweets, stratified by super-community. The blue line at the center is the average score, surrounded by the standard deviation range. Subjectivity ranges from 0 (very objective) to 1 (very subjective).

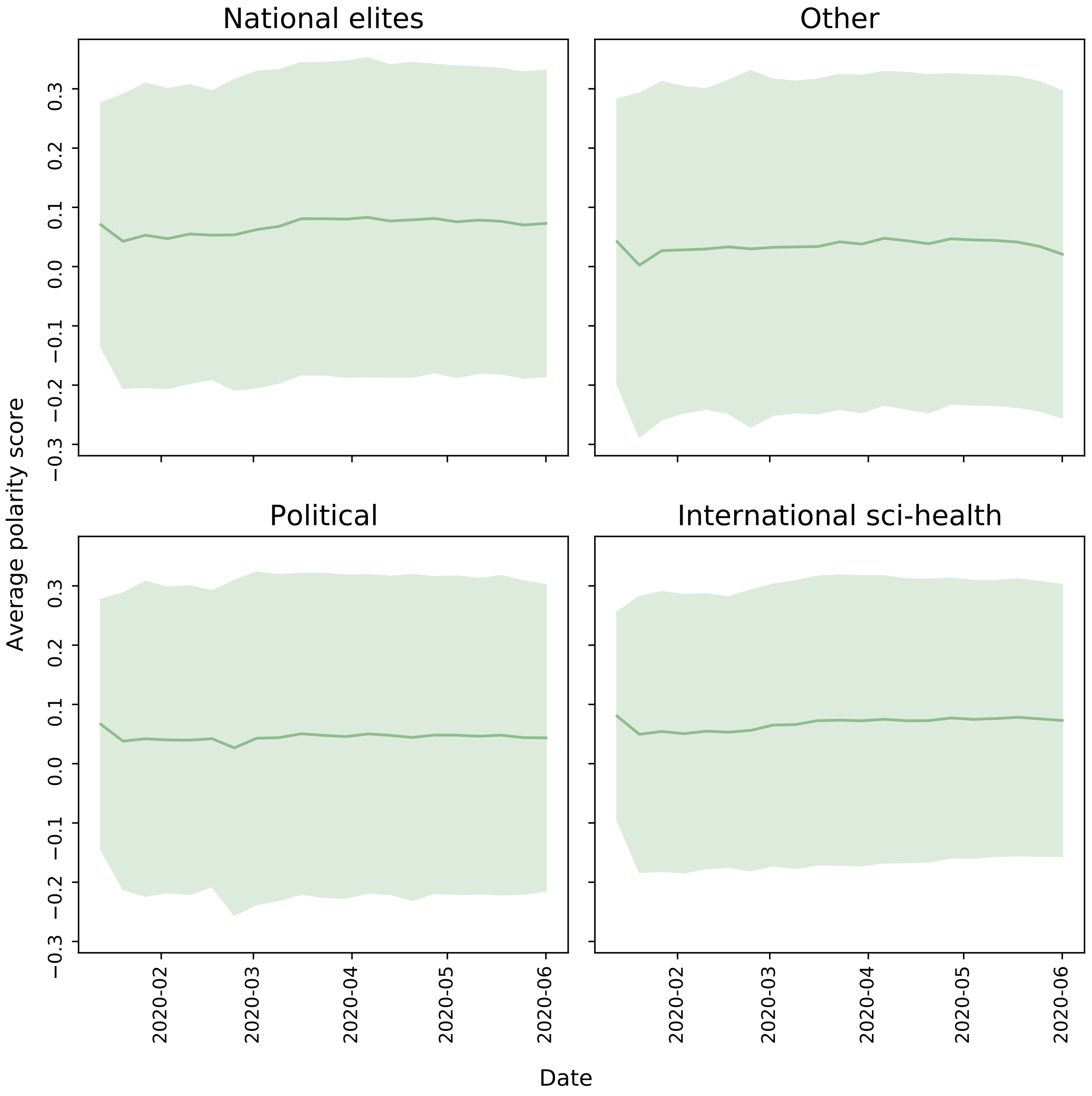

Figure 13: **Polarity score of tweets content.** Polarity score time-series for the original tweets, stratified by super-community. The green line at the center is the average score, surrounded by the standard deviation range. Polarity ranges from -1 (negative sentiment) to 1 (positive sentiment).

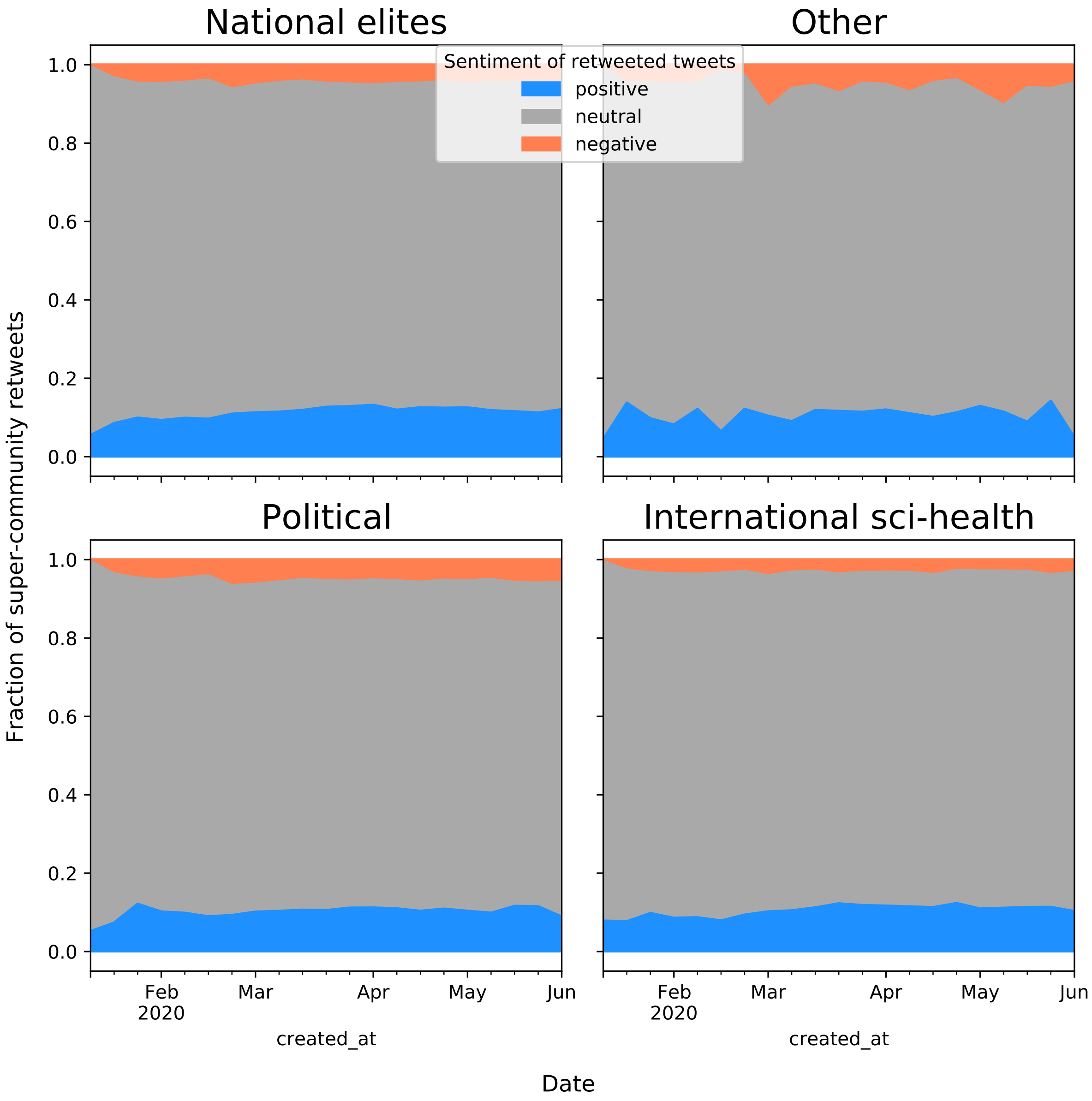

Figure 14: **Sentiment time-series of the retweets.** For each retweeting super-community, we show the time-series of the fraction of retweets to Negative, Neutral and Positive original tweets.

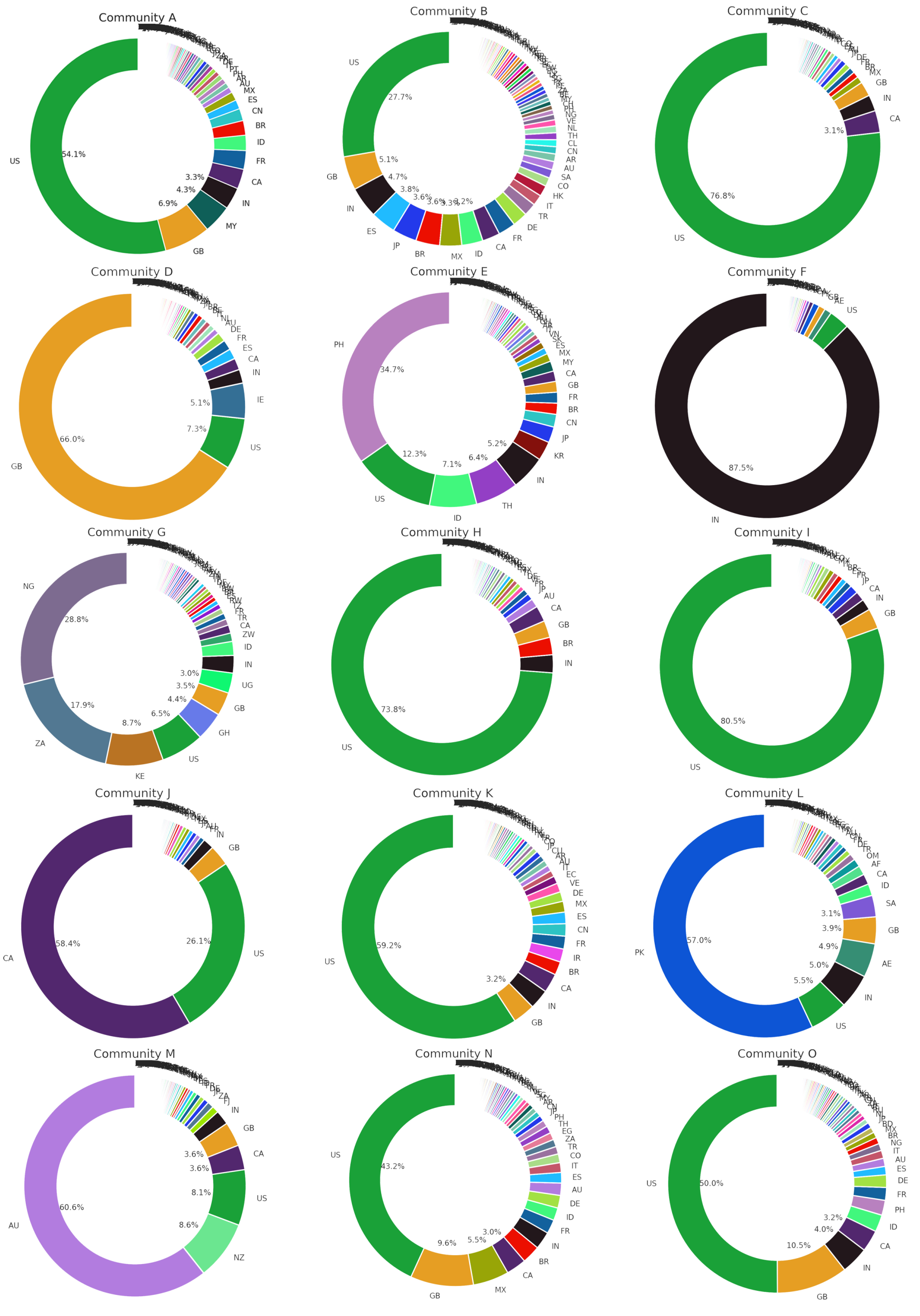

Figure 15: **Pie chart of the location of users, at country level.** Each user was assigned to the country code mostly represented in its tweets. Percent value is shown only for countries recurring more than 3% in the community users.

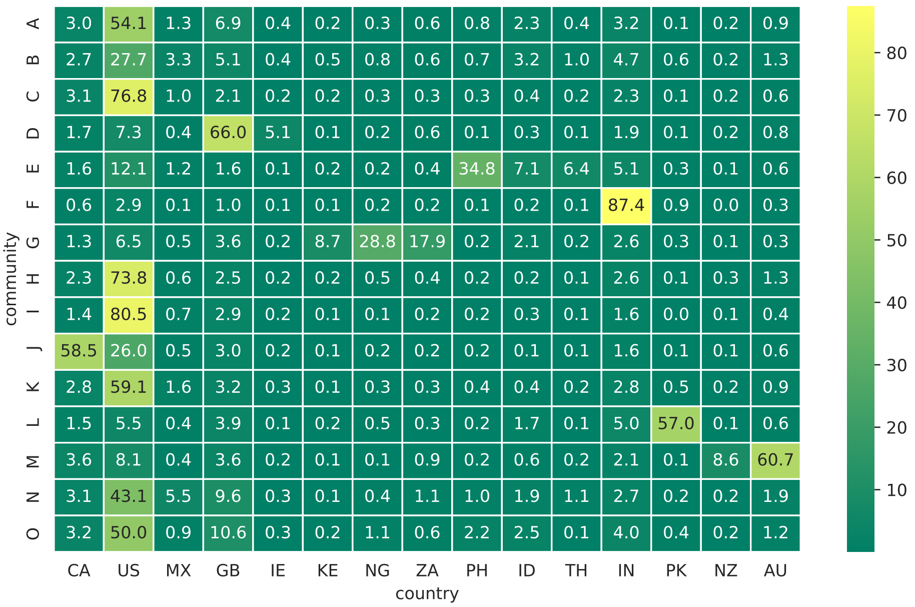

Figure 16: **Heatmap of community locations.** Each user was assigned to the country code mostly represented in its tweets. Only country codes represented at least by 5% in a community are displayed.

3 Supplementary Tables

Table 1: Overview of key properties of the 15 largest communities detected in the retweet network. Community name is ordered alphabetically by increasing size. 2nd largest category was reported in the table, since category "Other" was the most abundant on for all the communities. Majority location was explicitly reported only when exceeding 50%, indicating "int." for *international* otherwise.

**Community**	**2nd largest user category**	**Majority location**	**Number of users**	**Super-community**
A	Arts & Entertainment (3.3%)	US	7,464,665 (33.3%)	Other
B	Science (9.7%)	int.	2,366,768 (10.6%)	International sci-health
C	Political Supporter (6.2%)	US	2,231,259 (10.0%)	Political
D	Science (9.3%)	GB	2,117,691 (9.4%)	National elite
E	Arts & Entertainment (1.0%)	int.	1,616,006 (7.2%)	Other
F	Science (6.5%)	IN	1,538,840 (6.9%)	Political
G	Science (8.4%)	int.	1,436,377 (6.4%)	International sci-health
H	Political Supporter (12.3%)	US	1,217,933 (5.4%)	Political
I	Sports (10.7%)	US	465,125 (2.1%)	National elite
J	Science (9.9%)	CA	456,399 (2.0%)	National elite
K	Arts & Entertainment (5.9%)	US	423,077 (1.9%)	Political
L	Science (6.9%)	PK	252,111 (1.1%)	Political
M	Science (13.8%)	AU	186,216 (0.8%)	National elite
N	Adult content (18.7%)	US	133,771 (0.6%)	Other
O	Business (3.1%)	US	124,110 (0.6%)	Other

Table 2: Keywords used to collect data on the Twitter filter stream. Keywords used represent the way the sars-cov-2 virus was referred to at different points in time.

Date of change	Keywords
2020-01-13	wuhan
2020-01-14	wuhan, ncov
2020-01-21	wuhan, ncov, coronavirus
2020-02-11	wuhan, ncov, coronavirus, covid
2020-02-18	wuhan, ncov, coronavirus, covid, sars-cov-2
